# Supplementary material for: Iterative improvement in the automatic modular design of robot swarms
Source: PeerJ Comput Sci. 2020 Dec 7;6:e322. doi: 10.7717/peerj-cs.322 (PMC7924708; doi:10.7717/peerj-cs.322)
Supplement: Supplemental Information 3 [file peerj-cs-06-322-s003.zip › argos3/doc/api/standalone/a00589_source.html]

ARGoS: plugins/simulator/entities/rab\_equipped\_entity.h Source File


- Main Page
- Related Pages
- Namespaces
- Classes
- Files

- File List
- File Members

# plugins/simulator/entities/rab\_equipped\_entity.h

Go to the documentation of this file.

```
00001 
00007 #ifndef RAB_EQUIPPED_ENTITY_H
00008 #define RAB_EQUIPPED_ENTITY_H
00009 
00010 namespace argos {
00011    class CRABEquippedEntity;
00012    class CEmbodiedEntity;
00013    struct SAnchor;
00014 }
00015 
00016 #include <argos3/core/utility/datatypes/byte_array.h>
00017 #include <argos3/core/utility/datatypes/set.h>
00018 #include <argos3/core/simulator/entity/positional_entity.h>
00019 #include <argos3/core/utility/math/vector3.h>
00020 #include <argos3/core/simulator/space/positional_indices/space_hash.h>
00021 #include <argos3/core/simulator/space/positional_indices/grid.h>
00022 
00023 namespace argos {
00024 
00025    class CRABEquippedEntity : public CPositionalEntity {
00026 
00027    public:
00028 
00029       ENABLE_VTABLE();
00030 
00031       typedef std::vector<CRABEquippedEntity*> TVector;
00032       typedef CSet<CRABEquippedEntity*> TSet;
00033 
00034    public:
00035 
00036       CRABEquippedEntity(CComposableEntity* pc_parent);
00037 
00038       CRABEquippedEntity(CComposableEntity* pc_parent,
00039                          const std::string& str_id,
00040                          size_t un_msg_size,
00041                          Real f_range,
00042                          SAnchor& s_anchor,
00043                          CEmbodiedEntity& c_entity_body,
00044                          const CVector3& c_position = CVector3(),
00045                          const CQuaternion& c_orientation = CQuaternion());
00046 
00047       virtual void Init(TConfigurationNode& t_tree);
00048 
00049       virtual ~CRABEquippedEntity() {}
00050 
00051       virtual void Reset();
00052 
00053       virtual void Enable();
00054 
00055       virtual void Disable();
00056 
00057       virtual void Update();
00058 
00059       inline CEmbodiedEntity& GetEntityBody() {
00060          return *m_pcEntityBody;
00061       }
00062 
00063       inline size_t GetMsgSize() const {
00064          return m_cData.Size();
00065       }
00066 
00067       inline CByteArray& GetData() {
00068          return m_cData;
00069       }
00070 
00071       void SetData(const CByteArray& c_data);
00072 
00073       void ClearData();
00074 
00075       inline Real GetRange() const {
00076          return m_fRange;
00077       }
00078 
00079       inline void SetRange(Real f_range) {
00080          m_fRange = f_range;
00081       }
00082 
00083       inline const SAnchor& GetAnchor() const {
00084          return *m_psAnchor;
00085       }
00086 
00087       virtual std::string GetTypeDescription() const {
00088          return "rab";
00089       }
00090 
00091    protected:
00092 
00093       SAnchor* m_psAnchor;
00094       CVector3 m_cPosOffset;
00095       CQuaternion m_cRotOffset;
00096       CByteArray m_cData;
00097       Real m_fRange;
00098       CEmbodiedEntity* m_pcEntityBody;
00099 
00100    };
00101 
00102    /****************************************/
00103    /****************************************/
00104 
00105    class CRABEquippedEntitySpaceHashUpdater : public CSpaceHashUpdater<CRABEquippedEntity> {
00106 
00107    public:
00108 
00109       virtual void operator()(CAbstractSpaceHash<CRABEquippedEntity>& c_space_hash,
00110                               CRABEquippedEntity& c_element);
00111 
00112    private:
00113 
00114       SInt32 m_nCenterI, m_nCenterJ, m_nCenterK;
00115 
00116    };
00117 
00118    /****************************************/
00119    /****************************************/
00120 
00121    class CRABEquippedEntityGridCellUpdater : public CGrid<CRABEquippedEntity>::CCellOperation {
00122 
00123    public:
00124 
00125       CRABEquippedEntityGridCellUpdater(CGrid<CRABEquippedEntity>& c_grid);
00126 
00127       virtual bool operator()(SInt32 n_i,
00128                               SInt32 n_j,
00129                               SInt32 n_k,
00130                               CGrid<CRABEquippedEntity>::SCell& s_cell);
00131 
00132       void SetEntity(CRABEquippedEntity& c_entity);
00133 
00134    private:
00135 
00136       CGrid<CRABEquippedEntity>& m_cGrid;
00137       CRABEquippedEntity* m_pcEntity;
00138    };
00139 
00140    class CRABEquippedEntityGridEntityUpdater : public CGrid<CRABEquippedEntity>::COperation {
00141 
00142    public:
00143 
00144       CRABEquippedEntityGridEntityUpdater(CGrid<CRABEquippedEntity>& c_grid);
00145       virtual bool operator()(CRABEquippedEntity& c_entity);
00146 
00147    private:
00148 
00149       CGrid<CRABEquippedEntity>& m_cGrid;
00150       CRABEquippedEntityGridCellUpdater m_cCellUpdater;
00151    };
00152 
00153    /****************************************/
00154    /****************************************/
00155 
00156 }
00157 
00158 #endif
```

---

Generated on 10 Jul 2018 for ARGoS by 
 1.6.1
